# Clinical analysis of preoperative risk factors for the incidence of deep venous thromboembolism in patients undergoing posterior lumbar interbody fusion

**DOI:** 10.1186/s13018-016-0403-0

**Published:** 2016-06-13

**Authors:** Jingchao Wei, Wenyi Li, Yueying Pei, Yong Shen, Jia Li

**Affiliations:** Department of Orthopaedic Surgery, The Third Hospital of Hebei Medical University, 139 Ziqiang Road, Shijiazhuang, 050051 People’s Republic of China; The Key Laboratory of Orthopedic Biomechanics of Hebei Province, The Third Hospital of Hebei Medical University, Shijiazhuang, 050051 People’s Republic of China; Department of Orthopedic Surgery, Hebei General Hospital, 348 Heping Road, Shijiazhuang, 050000 People’s Republic of China; Department of ultrasound, Hebei General Hospital, 348 Heping Road, Shijiazhuang, 050000 People’s Republic of China

**Keywords:** Deep venous thrombosis, Posterior lumbar interbody fusion, Preoperative risk factors, Preoperative plasma D-dimer level

## Abstract

**Background:**

The purpose of this study aimed to assess preoperative risk factors for the incidence of deep venous thromboembolism in patients undergoing posterior lumbar interbody fusion (PLIF).

**Methods:**

The diagnosis of preoperative deep vein thrombosis (DVT) was confirmed by Doppler ultrasonography. To examine the preoperative risk factors for DVT admitted for PLIF, comparative analysis of the DVT-positive and DVT-negative groups was done.

**Results:**

DVT was detected in 9.4 % (269/2861) patients, including 17 proximal DVT patients (6.3 %) and 252 the distal DVT patients (93.7 %). According to multivariate logistic regression analysis, the age, preoperative D-dimer, and history of rheumatoid arthritis were significant risk factors relative to the onset of DVT after posterior lumbar surgery.

**Conclusions:**

According to the result of our study, age, positive preoperative plasma D-dimer level, and rheumatoid arthritis had the influential impact on the incidence of DVT admitted for PLIF.

## Background

Venous thromboembolism (VTE) is a common complication of spinal surgery which included deep vein thrombosis (DVT) and pulmonary embolism (PE). Many studies had reported the risk factors for DVT development in patients who undergo spine surgery, such as advanced age, obesity, neurological deficit, hospitalization, major surgery, immobilization, blood transfusion, malignancy, and trauma. Risk factors of DVT were common in patients with degenerative spine diseases [1–3]. Without prophylaxis, approximately 15 % of patients undergoing posterior spinal surgery develop DVT [4].

The need for appropriate prophylaxis was increasingly accepted. There were variety of strategies, including mechanical and chemical prophylaxis, which had been shown to be effective in preventing postoperative VTE [5–7]. The low molecular weight heparin (LMWH) had been used as chemical prophylaxis in decreasing the incidence of VTE in patients after spinal surgery. However, there was still got the fear of hemorrhagic disorders and spinal epidural hematoma (SEH) [8]. Therefore, it was still a matter of debate about the choice of prophylaxis in patients following spinal surgeries regardless of these strategies were used to prevent VTE.

D-dimer was widely used to diagnose VTE and markedly high in comparison to those in healthy volunteer [9]. However, there was insufficient published data focus on whether patients with preoperative plasma D-dimer level needed to use preoperative chemical prophylaxis. Furthermore, the efficacy of using preoperative LMWH on incidence of VTE or hemorrhagic disorders and SEH after surgery was still unknown. The purpose of this study aimed to assess the effect of preoperative plasma D-dimer level on the incidence of deep venous thromboembolism in patients undergoing posterior lumbar interbody fusion (PLIF).

## Methods

This study retrospectively reviewed of patients who underwent posterior lumbar interbody fusion, in the authors’ center between March 2008 and November 2014. Patients were diagnosed with lumbar pathologies such as lumbar disc herniation, lumbar spinal stenosis, and isthmic spondylolisthesis, by clinical and physical examinations corresponded to manifestations of lumbar spine radiographs, computed tomography (CT) scans, and magnetic resonance images (MRI). Patients who had preoperative urinalysis positive for red blood cells, fecal occult blood, skin purpura, or hematoma; fracture, infection, or tumor; active bleeding or high risk of bleeding; oral anticoagulant therapy 1 week prior to the surgery; and LMWH hypersensitivity were excluded from this study. Those patients with a previous history of DVT or the existence of DVT was detected preoperatively were also excluded from this study. The preoperative plasma D-dimer level was defined before the study as a plasma concentration of 0.50 μg/ml or more, as recommended by the manufacturer.

All patients received mechanical prophylaxis (intermittent pneumatic compression devices) from induction of general anesthesia to postoperative ambulation. The patients were routinely administered daily LMWH (Enoxaparin, 40 mg/day) from postoperative day (POD) 1 to POD 7. For the patients with preoperative positive value of D-dimer, the first subcutaneous injection was given 12 h prior to surgery. Walking exercise started 5 days after surgery.

Both groups had a daily physical evaluation in order to find early onset DVT symptoms and symptomatic epidural/wound hematoma during hospitalization. Doppler ultrasonography was performed on patients within 24 h of admission to the hospital, and the second examination were performed 5 days postoperatively. In cases where DVT symptoms (warmth, erythema, unilateral calf pain, edema) were detected after the operation, an additional Doppler ultrasonography was performed on the patient.

This study was approved by the Institutional Review Board of the Third Hospital of Hebei Medical University. Subjects provided informed consent to participate in the study; those who did not wish to participate in this study were not enrolled.

### Statistical analysis

Statistical analysis was performed with SPSS 17.0 for Windows (SPSS Inc., Chicago, IL, USA). The patients’ characteristics between groups were compared using the Mann-Whitney *U* test and Fisher’s exact test. After these univariate analyses, a *P* < 0.2 was selected and evaluated by multivariate logistic regression analysis to identify predictors of DVT among independent variable. Conclusively, a *P* < 0.05 was considered statistically significant.

## Results

In this retrospective study, 3629 patients initially identified, a total of 2861 patients were admitted into analyses, and 768 were out for adequate clinical information in the medical record. According to the incidence of DVT, 269 patients were classified into DVT-positive group and 2592 patients comprised DVT-negative group. In this series, patients completed the study without any life-threatening complications. There were no patients with clinical signs of DVT or PE. However, 6 cases (0.21 %) developed SEH in 2 days after surgery.

Of 269 DVT-positive patients, the distal thrombus was identified in 252 (93.7 %); the proximal thrombus only was identified in 17 (6.3 %). The incidence of DVT was significantly higher in the preoperative D-dimer-positive patients compared to the D-dimer-negative patients (*P* = 0.000). The mean age in DVT-positive group was significantly older than that in DVT-negative patients (*P* = 0.002).

The most frequent preoperative medical history in DVT-positive group was hypertension (137 patients, 50.9 %); the second was coronary heart disease (102 patients, 37.9 %). Rheumatoid arthritis was seen in 0.5 % (12/2592) of the DVT-negative group and 11.5 % (31/269) of the DVT-positive group (*P* < 0.05). There were 32.0 % (82/269 patients) of the DVT-positive group who had undergone major surgery, but only 25.0 % (648/2592) of the DVT-negative group had undergone major surgery (*P* = 0.006) (Table 1).

**Table 1:** Univariate analysis of the risk factors for DVT

Characteristic	DVT (+) group	DVT (−) group	*P* value
No. of patients	269	2595	
Age	61.3 ± 10.3	52.6 ± 11.7	0.002
Gender(M/F)	129/140	1382/1213	0.097
BMI (kg/m^2^)	24.2 ± 3.6	23. 5 ± 2.9	0.376
Hypertension	137	1509	0.066
Coronary heart disease	102	956	0.301
Diabetes mellitus	89	823	0.633
Rheumatoid arthritis	31	12	0.000
Major surgery	82	648	0.164
Lumbar pathologies			0.659
Lumbar disc herniation	87	901	
Lumbar spinal stenosis	107	1026	
Isthmic spondylolisthesis	75	668	
Preoperative D-dimer (±)	112/157	89/2056	0.000

To compare the relative impact of these variables on the incidence of DVT, multiple logistic regression analysis was performed. Statistically significant risk factors for DVT included preoperative plasma D-dimer level, age, hypertension, rheumatoid arthritis, and major surgery in the univariate analysis. The multivariate analysis showed that preoperative plasma D-dimer level, age, and rheumatoid arthritis were significant independent risk factors for DVT admitted PLIF (Table 2).

**Table 2:** Multivariate analysis of the risk factors for DVT

Risk factors	*P*	OR	95 % CI
Age	0.021	1.0261	0.9810–1.0643
Gender	0.298	0.1833	0.0043–5.2853
Hypertension	0.156	1.5422	0.9853–0.9368
Preoperative D-dimer	0.013	6.2217	1.0354–2.9310
Major surgery	0.113	0.1512	0.0694–0.9393
Rheumatoid arthritis	0.003	6.1203	1.0412–42.9899

## Discussion

As we all know, PLIF was likely to be associated with postoperative DVT in spinal surgery. The findings of our study indicated that although demographics and basic characteristics associated with DVT-positive and DVT-negative group were generally similar, DVT-positive was associated with a significantly high preoperative plasma D-dimer level compared to the DVT-negative group. According to the chronic disease history, patients with rheumatoid arthritis were significantly high incidence in DVT group. It also provided evidence for the safety of LMWH at preoperative and postoperative in PLIF. The multivariate analysis showed that preoperative plasma D-dimer level, age (*P* < 0.013), and rheumatoid arthritis (*P* = 0.028) were significant independent preoperative risk factors for DVT admitted for PLIF.

D-dimer testing had been widely used in the exclusion of DVT/PE. A positive D-dimer test usually indicates blood clots or thrombosis in the body, which is associated with the severity of DVT. A standard cutoff of 0.50 μg/ml was used in routine clinical practice, with values above that level considered positive. However, the customary D-dimer cutoff is inappropriate under different conditions, including infection and pregnancy. Previous studies had shown that D-dimer level of patients undergoing surgical procedures would result from the development of DVT [9]. D-dimer level was a negative predictor for DVT which was helpful for diagnostic aid in suspected DVT, however, with a not very high sensitivity as it had been reported [10]. Whether an elevated level of D-dimer before PLIF was associated with the occurrence of postoperative DVT has not been definitely established and warrants further investigation. In this study, the incidence of DVT was significantly higher in the preoperative D-dimer positive patients compared to the negative patients (*P* < 0.05). The multivariate analysis showed the preoperative plasma D-dimer level was significant independent preoperative risk factors for DVT admitted for PLIF.

Reducing the risk of VTE in patients undergoing spinal surgery, it had recommended combined mechanical and chemical prophylaxis. Oda et al. had found the prevalence of DVT after posterior spinal surgery without prophylaxis was 15.5 % which was higher than generally recognized [4].

The type of common prophylactic treatment after spinal surgery had been used including mechanical (intermittent pneumatic compression) and chemical prophylaxis (LMWH). The use of LMWH was the most effective methods, which was preferred by most surgeons [8, 11, 12]. However, there was still controversy over the effect of LMWH prophylaxis in spinal surgery because of the fear of SEH and bleeding complications. Glotzbecker et al. had compared the effect of mechanical prophylaxis with chemical prophylaxis after spinal surgery. It had suggested that the use of mechanical prophylaxis could be a primary method to decrease the incidence of DVT. Furthermore, the chemical prophylaxis was associated with the lowest prevalence of DVT incidence after spinal surgery and no evidence to support or refute the use of chemical anticoagulants in routine elective spinal surgery [13]. Strom et al. retrospectively reviewed cases of cervical and lumbar laminectomy and found low hemorrhage risk with LMWH prophylaxis after spinal surgery. In their opinion, patients undergoing laminectomy for degenerative disease without LMWH prophylaxis might be at significant risk for DVT[7]. Gerlach et al. reported the results of 1299 patients who underwent lumbar surgery. To prevent DVT, all patients were routinely treated subcutaneously with mechanical and chemical prophylaxis until discharge. Their results confirmed that early postoperative chemical prophylaxis in patients with lumbar surgery without an increased risk of postoperative hemorrhage [14]. This study assessed the effect of LMWH on positive preoperative plasma D-dimer level patients undergoing PLIF. From this study, we could conclude that probably, medication of LMWH as a prophylaxis has not adversely significant hemorrhagic or SEH. For preoperative positive value of D-dimer patients, combinations of mechanical and chemical prophylaxis were strongly recommended.

Although statistical support for spine-specific risk factors was lacking, several studies had noted common observations for patients who sustained a DVT. Previous studies had demonstrated the risk factors associated with DVT after spinal surgery, including advanced age, D-dimer, previous thromboembolism, ratio of perioperative immobilization, type of spinal surgery, and hypertension. Sansone et al. analyzed a total of 4383 patients who had undergone elective spine surgery. In their study, the risk of DVT was relatively low, especially for the patients who receive chemical prophylaxis. Unfortunately, chemical prophylaxis exposes patients to a greater risk of hemorrhagic disorders and SHE [15]. Yoshioka et al. reported the statistically significant risk factors for DVT including advanced age, neurologic deficits, and spinal tumor. Of these factors, spinal tumor was a high risk of DVT. In another study, the fibrin monomer complex measured 1 day after surgery was considered to be useful as an indicator of DVT [16, 17]. Oda et al. found a statistically significant risk of VTE associated with advanced age and anatomic location of surgery [4].

In the present study, the results indicated that more patients with a positive preoperative plasma D-dimer level in DVT group than non-DVT group. However, there was no significant difference among the lumbar pathologies. Therefore, it is believed that the lumbar pathology itself had not a higher impact on the incidence of VTE. Univariate analysis found that positive preoperative plasma D-dimer level, advanced age, history of rheumatoid arthritis, hypertension, and major surgery tended to be risk factors for DVT. According to the multivariate logistic regression analysis, it had shown that positive preoperative plasma D-dimer level, advanced age, and history of RA were independent risk factors for postoperative DVT in patients admitted for PLIF.

Previous studies have found elevated risks of DVT in patients with rheumatoid arthritis undergoing joint arthroplasty [18–20]. It had been confirmed that patients with rheumatoid arthritis had an increased risk of VTE compared to the general population. According to this study, the prevalence of postoperative DVT was significantly higher in rheumatoid arthritis patients admitted for PLIF than others. Patients with rheumatoid arthritis got an increased risk of developing DVT, which might be attributable to the presence of systemic inflammatory disease. Moreover, the use of corticosteroids might accelerate atherosclerotic disease.

### Limitation

However, there were several limitations in this study. Owing to insufficient information, this study could not possible to give a suggestion on standardized preoperative chemical prophylaxis protocol. Whether mechanical prophylaxis alone or combined with chemical prophylaxis could be efficacious on the positive preoperative plasma D-dimer level is unknown. Further studies are warranted to investigate preoperative plus postoperative prophylaxis for patients undergoing other spinal surgeries. Furthermore, it must be investigated the necessity of the use of LMWH prophylaxis, its initiation time and length, particularly in high-risk patients. A prospective multiple-center study are needed to assess the safety of early LMWH administration which would certainly provide more useful information. In the future, we will further investigate the relationship between RA associated D-dimer increase and DVT.

## Conclusions

According to the result of our study, age, positive preoperative plasma D-dimer level, and rheumatoid arthritis had the influential impact on the incidence of DVT admitted for PLIF. For positive preoperative plasma D-dimer level patients, combinations of mechanical and chemical prophylaxis were recommended. Surgeons should be pay attention to decrease the incidence of DVT if specific preoperative risk factors were present.
